# Radiomic Model for Determining the Value of Elasticity and Grayscale Ultrasound Diagnoses for Predicting BRAF^V600E^ Mutations in Papillary Thyroid Carcinoma

**DOI:** 10.3389/fendo.2022.872153

**Published:** 2022-04-22

**Authors:** Yu-guo Wang, Fei-ju Xu, Enock Adjei Agyekum, Hong Xiang, Yuan-dong Wang, Jin Zhang, Hui Sun, Guo-liang Zhang, Xiang-shu Bo, Wen-zhi Lv, Xian Wang, Shu-dong Hu, Xiao-qin Qian

**Affiliations:** ^1^ Department of Ultrasound, Jiangsu Hospital of Integrated Traditional Chinese and Western Medicine, Nanjing, China; ^2^ Department of Ultrasound, Affiliated People’s Hospital of Jiangsu University, Zhenjiang, China; ^3^ Department of Pediatrics, Affiliated Hospital of Jiangsu University, Zhenjiang, China; ^4^ Department of Radiotherapy, Affiliated People’s Hospital of Jiangsu University, Zhenjiang, China; ^5^ Department of Pathology, Affiliated People’s Hospital of Jiangsu University, Zhenjiang, China; ^6^ Department of General Surgery, Affiliated People’s Hospital of Jiangsu University, Zhenjiang, China; ^7^ Department of Artificial Intelligence, Julei Technology, Company, Wuhan, China; ^8^ Department of Radiology, Affiliated Hospital of Jiangnan University, Wuxi, China

**Keywords:** ultrasonic examination, radiomic, papillary thyroid carcinoma, BRAF-V600E, model

## Abstract

BRAF^V600E^ is the most common mutated gene in thyroid cancer and is most closely related to papillary thyroid carcinoma(PTC). We investigated the value of elasticity and grayscale ultrasonography for predicting BRAF^V600E^ mutations in PTC.

**Methods:**

138 patients with PTC who underwent preoperative ultrasound between January 2014 and 2021 were retrospectively examined. Patients were divided into BRAF^V600E^ mutation-free group (n=75) and BRAF^V600E^ mutation group (n=63). Patients were randomly divided into training (n=96) and test (n=42) groups. A total of 479 radiomic features were extracted from the grayscale and elasticity ultra-sonograms. Regression analysis was done to select the features that provided the most information. Then, 10-fold cross-validation was used to compare the performance of different classification algorithms. Logistic regression was used to predict BRAF^V600E^ mutations.

**Results:**

Eight radiomics features were extracted from the grayscale ultrasonogram, and five radiomics features were extracted from the elasticity ultrasonogram. Three models were developed using these radiomic features. The models were derived from elasticity ultrasound, grayscale ultrasound, and a combination of grayscale and elasticity ultrasound, with areas under the curve (AUC) 0.952 [95% confidence interval (CI), 0.914−0.990], AUC 0.792 [95% CI, 0.703−0.882], and AUC 0.985 [95% CI, 0.965−1.000] in the training dataset, AUC 0.931 [95% CI, 0.841−1.000], AUC 0. 725 [95% CI, 0.569−0.880], and AUC 0.938 [95% CI, 0.851−1.000] in the test dataset, respectively.

**Conclusion:**

The radiomic model based on grayscale and elasticity ultrasound had a good predictive value for BRAF^V600E^ gene mutations in patients with PTC.

## Introduction

Papillary thyroid carcinoma (PTC) is the most common type of pathological thyroid cancer, accounting for approximately 80–90% of all thyroid cancers and with an annually increasing incidence (1, 2). Most patients have a low death rate, with a 10-year survival rate of > 90%; however, some histological subtypes are prone to extraglandular thyroid invasion, vascular invasion, a high postoperative recurrence rate, and distant metastasis rate. The BRAF^V600E^ gene encodes a protein-dependent kinase, an essential component of the mitogen-activated protein kinase pathway that plays a crucial role in the regulation of cell proliferation, differentiation, and apoptosis (3, 4). The BRAF^V600E^ gene has the highest mutation rate in thyroid cancer and is closely associated with PTC. The mutation of BRAF^V600E^ is an important factor in the PTC phenotype, which contributes to the diagnosis and differential diagnoses of PTC; additionally, the detection rate of PTC is 30–84% (4, 5). The BRAF^V600E^ mutation is closely related to PTC extrathyroid extension invasion and cervical lymph node metastasis, suggesting an invasion of patients with PTC (6, 7). The BRAF^V600E^ mutation was also included in the risk stratification of PTC in the 2015 American Thyroid Association Guidelines; therefore, the preoperative diagnosis of the BRAF^V600E^ mutation is closely related to the development of an appropriate surgical treatment plan to prevent the recurrence of PTC.

While recent studies have shown that grayscale ultrasound can predict the mutation of BRAF^V600E^ in PTC, the results are highly controversial (8, 9). Kabaker et al. reported that vertical and horizontal diameter ratios >1, an unclear boundary, low echo, calcification, and no halo were all associated with the BRAF^V600E^ mutation on ultrasound examination (10). Similarly, Hahn et al. reported that vertical and horizontal diameter ratios >1 were associated with BRAF^V600E^ gene mutations (11); however, some related scholars simultaneously reported that there were no ultrasonic characteristics related to the BRAF^V600E^ mutation (12, 13). Ultrasound radiomics (USR) is a branch of radiomics that is based on ultrasound examination, combined with genetic and clinical data, and employs artificial intelligence as a tool for high-throughput extraction and analysis of relevant tumor information, from structure to molecular cell function analysis, identify tissue and cell level characteristics that cannot be detected by visual reading or conventional quantitative technology, and provide auxiliary decision support for clinical diagnosis and prognosis (14, 15). With high throughput, it can transform the image data from the region of interest (ROI) of lesions into quantitative data with feature space, performing precise quantitative analysis. Previous studies have shown that ultrasonic histogram and texture analysis can help distinguish between benign and malignant thyroid nodules (15, 16); however, there are few reports regarding the ultrasonic image features of the BRAF^V600E^ mutation in PTC using the USR method (8, 9), and there is no further application of multimodal USR.

In this study, the USR features of PTC were analyzed based on grayscale and elasticity ultrasound to predict the risk of the BRAF^V600E^ mutation.

## Materials and Methods

### Clinical Data

**Figure 1:**
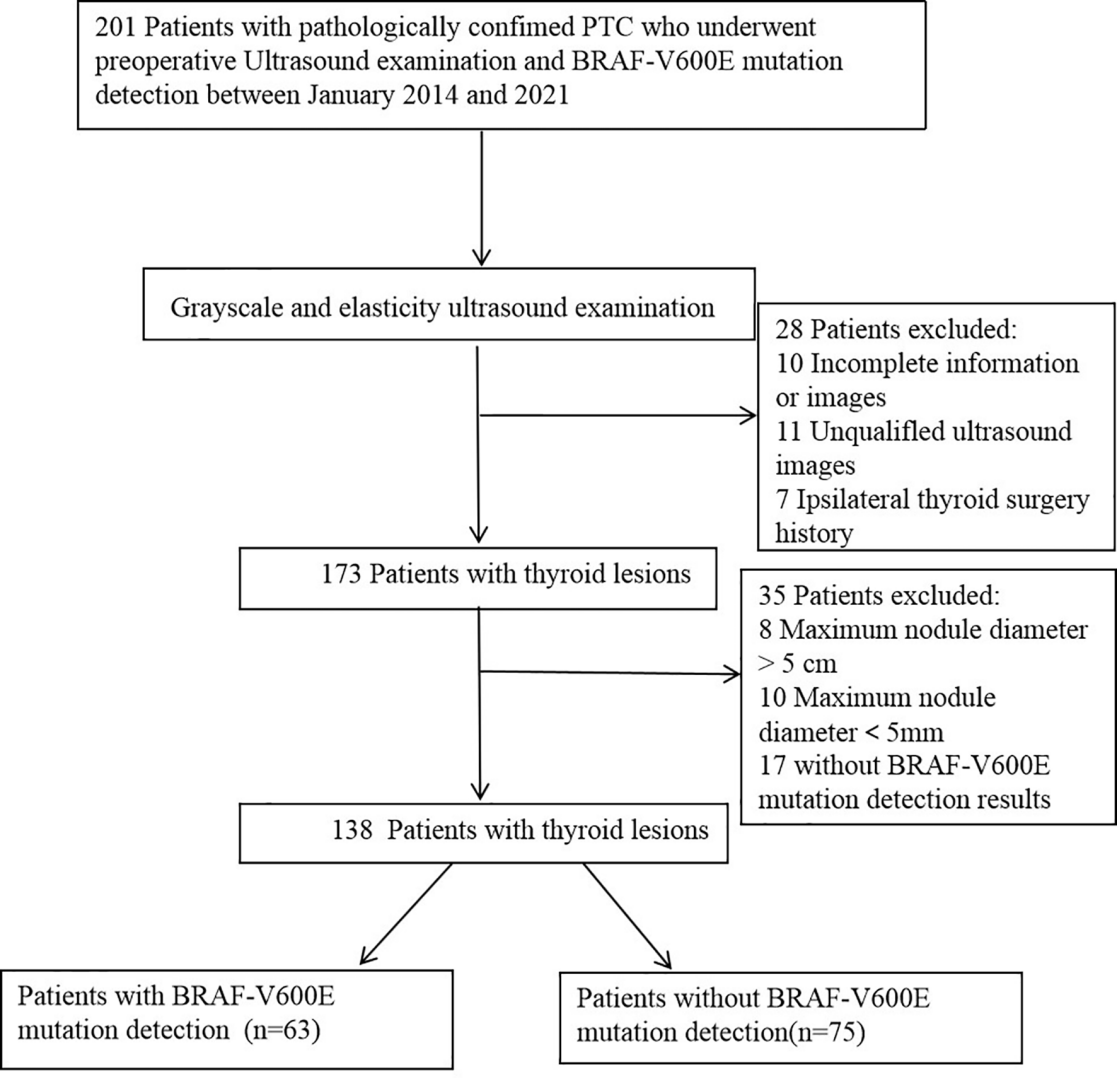
Schematic diagram of the patient selection. PTC, papillary thyroid carcinoma.

This study was approved by the Ethics Committee of Jiangsu University Affiliated People’s Hospital, and all patients provided written informed consent. PTC patients who underwent a preoperative thyroid ultrasound examination, BRAF^V600E^ mutation detection, and surgery at Jiangsu University Affiliated People’s Hospital between January 2014 and 2021, were retrospectively examined. **Figure 1** shows the enrolment procedure. The inclusion criteria were having undergone preoperative grayscale and elasticity ultrasound examination of the thyroid, with related ultrasound images and diagnostic results obtained; a maximum nodule diameter > 5 mm; a maximum nodule diameter > 5 mm, < 5 cm; postoperative pathology-confirmed PTC; and unilateral and single focal lesion. The exclusion criteria were unclear ultrasound images of nodules owing to artifacts and a maximum nodule diameter > 5 cm. Finally, 138 PTCs of 138 patients (mean age, 41.63 ± 11.36 [range, 25–65] years) were analyzed in this study. One hundred thirty-eight patients were divided into BRAF^V600E^ mutation-free group (n=75) and BRAF^V600E^ mutation group (n=63). The clinical information of the enrolled patients, including age, sex, nodule diameter, nodule location, nodule echo, nodule boundary, nodule internal and peripheral blood flow, nodule elastic grading, calcification, cervical lymph node metastasis, and BRAF^V600E^ mutation results were recorded.

### Ultrasound and Elastic Ultrasound Examination

The Philips Q5 (both Healthcare, Eindhoven, Netherlands) and the GE LOGIC E20 (GE Medical Systems, American General) ultrasonic instrument (L12-5 linear array probe, frequency: 10–14 MHz) were used. The patients were placed in the supine position, and longitudinal and transverse continuous scanning were performed to obtain longitudinal and transverse images of the thyroid nodules. A coexisting diagram including the nodule diameter, nodule location, nodule echo, nodule boundary, blood flow in and around the nodule, elastic grading of the nodule, calcification, and cervical lymph node metastasis was observed. The elastic imaging mode was enabled, and the position and size of the sampling frame were adjusted on the cross-sectional image. The nodules were positioned at the center of the elastic imaging region, with the ROI more than four times the size of the nodules. The probe was perpendicular to the nodule, and pressure was applied in a steady manner (range 1–2 mm, 1–2 times/s). When the linear strain hint graph (green spring) indicated stability, the freeze key was pressed to obtain an elastic image; the color change in the ROI was observed (green indicated soft; red indicated hard), and the hardness of the nodule was scored based on elasticity. The grading standard of the elastic image was as follows: 1 point, nodules, and surrounding tissues were completely green; 2 points, nodules were red and green (mostly green); 3 points, mixed red and green distribution (mainly red); 4 points, nodules were completely red; and 5 points, the red coverage was greater than that of the nodule.

### ROI Segmentation and Normalization

Thyroid ultrasound examinations were performed within one week before surgery. Two radiologists (with 9 and 10 years of experience in the diagnosis of thyroid diseases, respectively) blinded to the pathological and surgical records, completed the manual ultrasonic image segmentation independently. The ROIs were placed on the tumor mass without the surrounding tissue, the ROIs were manually sketched on each image using the ITK-SNAP software (http://www.itksnap.org). The tumor regions in the elasticity ultrasound images were sketched according to the grayscale images. Following the manual ROI segmentation, the z-score normalization method was used to standardize the grayscale and elasticity ultrasound images to obtain a standard normal distribution of image intensities.

### Radiomic Feature Extraction and Feature Selection

**Figure 2:**
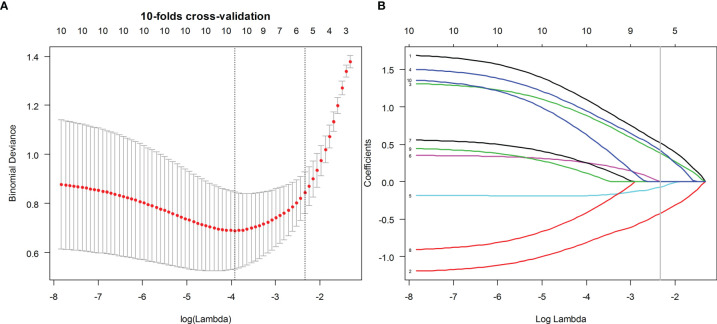
Ultrasound image feature selection using the least absolute shrinkage and selection operator (LASSO) logistic regression model in the training dataset. **(A)** The 10-fold cross-validation and the minimal criteria process were used to generate the optimal penalization coefficient lambda in the LASSO model. **(B)** LASSO coefficient profiles of the BMUS and ES features. The dotted vertical line was drawn at the value selected by 10-fold cross-validation when the optimal λ resulted in eight (BMUS) and five (ES) nonzero coefficients, respectively.

The PyRadiomics platform from the medical image data was used to extract the standardized set of radiology characteristics (https://github.com/Radiomics/pyradiomics). In total, 479 radiomics features were extracted from the ultrasound images of each ROI. The grayscale features were extracted from the ROI of the grayscale images, and elastic features were extracted from the ROI of the elastic images. The interclass correlation coefficient (ICC) was used to evaluate the inter-observer and intra-observer consistency of feature extraction. ICC > 0.80 was considered excellent. Spearman’s correlation analysis was used to select radiometric that were highly correlated with BRAF^V600E^ mutations. The redundant features identified by Spearman’s correlation coefficient ≥0.8 were eliminated; the minimum redundancy maximum relevance (mRMR) algorithm was subsequently used to select the top 10 features with high relevance and low redundancy for the following analyses. After mRMR, the least absolute shrinkage and selection operator (LASSO) logistic regression method, using 10-fold cross-validation, was used to select the most useful BRAF^V600E^ mutation-related predictive features in the training dataset (**Figure 2**).

According to the result of the LASSO regression analysis, the mathematical expression of the BMUS and ES radiomic signatures are given as follows:


BMUSRadiomicsignature=0.271−0.384×wavelet.LH_glrlm_RanVariance+0.224×wavelet.HH_glcm_Imc1+0.118×wavelet.HL_glcm_InverseVariance+0.086×wavelet.LH_glszm_SmallAreaEmphasis+0.058×wavelet.LL_glcm_MCC+0.055×wavelet.HH_glszm_SmallAreaLowGrayLevelEmphasis+0.51×wavelet.LH_firstorder_Median+0.025×wavelet.LH_glcm_Imc1

ESRadiomic signature =0.307+0.521×wavelet.HL_glszm_SmallAreaEmphasis−0.426×original_firstorder_Minimum+0.424×wavelet.HL_glcm_MCC+0.384×wavelet.HL_glrlm_HighGrayLevelRunEmphasis−0.073×original_glszm_GrayLevelNonUniformityNormalized

### Radiomic Model Construction and Validation

A single radiomics model using grayscale and elasticity ultrasound was generated using a linear combination of the selected features, weighted by the LASSO algorithm with non-zero coefficients. The combined grayscale and elasticity radiomics model was built based on multivariate analysis of the training dataset. Differences in the radiomics model between patients with and without the BRAF^V600E^ mutation were compared using the Mann–Whitney U test. The predictive performance of the radiomics model was evaluated using a receiver operating characteristic (ROC) curve, and the area under the curve (AUC), sensitivity, and specificity were calculated for both the training and test datasets.

### Detection Method of BRAF Gene

More than two needles were used to puncture tissue, and DNA was extracted using the QIAamp Human Genome DNA Extraction Kit (QIAGEN). The NanoDrop 1000 (Thermo Fisher, USA) was used to measure DNA concentrations, which were ≥50ng/l. The PCR reaction system was set up in accordance with the PCR kit’s specifications, which included a special probe, reaction solution, and pure water. The processed nucleic acid to be tested, as well as the positive and negative quality controls, were then added to the reaction system, centrifuged at 2000 rpm for 15 seconds to the bottom of the tube, and the PCR amplification reaction was carried out. The reaction conditions were set in accordance with the kit’s specifications. Following the completion of the reaction, the negative quality control should have no S-type amplification curve and the positive quality control should have an S-type amplification curve and a Ct value of 30. The results of the BRAF gene to be tested are finally read after the above two conditions are met.

### Statistical Analysis

Statistical analyses were performed using R software (version 3.6.1, https://www.r-project.org) and SPSS 20.0 software (SPSS,Inc., Chicago,IL). Descriptive statistics of continuous variables were expressed as mean ± standard deviation, and the age difference between the two groups was tested using the Student’s t-test. The χ2 test showed that the gender difference between the two groups was statistically significant (P<0.05). To evaluate the best predictive models, AUCs in the ROC analysis were calculated.

## Results

### Clinical Characteristics

In 138 patients with PTC, 78 cases were classical type, 49 cases were follicular type, and 6 cases were diffuse sclerosis type. The mutation rate of BRAF gene was 45.6% (63/138), 62.8% (49/78) and 16.3% (8/49). The mutation rate of diffuse sclerosing type was 33.3% (2/6) and that of hypercellular subtype was 80% (4/5). A total of 138 PTC patients, including 87 women (mean age, 41.81 ± 11.23 [range, 25–57] years) and 51 men (mean age, 43.82 ± 12.18 [range, 28–65] years). All patients were randomly divided into either the training group (n=96) or the test group (n=42) using a stratified sampling method, and the mutation and non-mutation groups were randomly divided at 7:3, respectively. A comparison of the clinical data and imaging comparisons of the 138 patients between the training and test groups is shown in **Table 1**. There were no significant differences in clinical characteristics between the two groups, including age and sex (P>0.05). There were also no significant differences in the mean nodule size (BRAF^V600E^ mutant group: 24.12 ± 8.6 mm; without BRAF^V600E^ mutant group: 23.98 ± 11.01 mm, P=0.928) or cervical lymph node metastasis (P=0.102) between the two groups. The relationship between the BRAF^V600E^ mutation and ultrasonic imaging characteristics is shown in **Table 2**.

**Table 1:** Clinical characteristics of the patients in the training and test cohorts.

Characteristic	Training cohort (n=96)	test cohort (n=42)	P-value
**Age, mean ± SD (years)**	41.78 ± 10.99	44.33 ± 12.81	0.152
**Age (years)**			
>45	48.63 ± 5.23	49.90 ± 5.89	0.218
≤45	34.61 ± 5.17	34.76 ± 7.87	0.670
**Sex**			
Male	36	15	0.851
Female	60	27	
**Tumor size(mm), mean ± SD**	26.04 ± 8.51	26.63 ± 8.55	0.074
**Primary site**			
Right lobe	28	15	0.318
Left lobe	30	16	
Isthmus	38	11	
**Tumor location**			
Upper pole	31	18	0.325
Lower pole	27	13	
Middle	38	11	
**Composition**			
Solid	56	19	0.269
Predominantly solid	40	23	
**Elastic classification**			
1	13	5	0.375
2	22	15	
3	15	7	
4	26	7	
5	20	8	
**Cystic change**			
With cystic change	52	20	0.579
Without cystic change	44	22	
**Calcification**			
Microcalcification	37	11	0.143
Macrocalcification	41	17	
Rim calcification	18	14	
**Tumor border**			
Clear	37	13	0.510
Less clear	31	18	
Fuzzy	28	11	
**Cervical lymph node metastasis**	61	31	0.327

**Table 2:** Relationship between BRAF mutations and US imaging characteristics through visual assessment of papillary thyroid carcinomas.

	BRAF mutation (n=63)	No BRAF mutation (n=75)	P-value
Age, mean ± SD, years	38.03 ± 10.41	36.68 ± 10.05	0.377
**Sex**			
Male	22	29	0.724
Female	41	46	
**Tumor size, mean ± SD**	24.12 ± 8.6	23.98 ± 11.01	0.928
**Composition**			
Solid	33	42	0.733
Predominantly solid	30	33	
**Solid part Echogenicity**			
Markedly hypoechoic	41	20	0.000
Hypoechoic	10	21	
Isoechoic	9	14	
Hyperechoic	3	20	
**Shape**			
Irregular	32	43	0.443
Round to oval	31	32	
**Vertical and horizontal diameter ratio**			
≥1	41	27	0.001
<1	22	48	
**Margin**			
Spiculated/microlobulated	30	25	0.183
Ill-defined	21	28	
Smooth	12	22	
**Calcification**			
Microcalcification	26	22	0.127
Macrocalcification	27	31	
Rim calcification	10	22	
**Final C-TIRADS category**			
Low suspicion	16	31	0.058
Intermediate suspicion	17	23	
High suspicion	30	22	
**Cervical lymph node metastasis**	47	45	0.102

### Feature Selection

**Figure 3:**
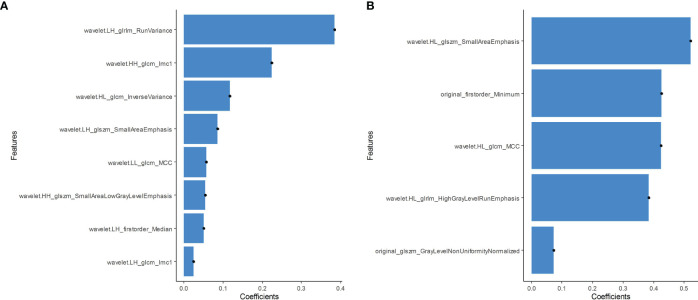
**(A)** Grayscale features were reduced to eight BRAF^V600E^ mutation-related features in the training cohort. **(B)** The elasticity features were reduced to five risk predictors in the training cohort.

In total, 479 radiomic features were extracted from the grayscale and elasticity ultra-sonograms of each patient. The grayscale features were reduced to eight BRAF^V600E^ mutation-related features after Spearman’s correlation analysis, mRMR, and LASSO algorithms were applied in the training cohort (**Figure 3**). Likewise, the elasticity features were reduced to five risk predictors in the training cohort (**Figure 3**). Favorable inter-and intra-observer reproducibility of feature extraction was achieved, with intra-observer ICCs of 0.487–0.965 and inter-observer ICCs of 0.543–0.982.

### Model Construction and Validation

**Figure 4:**
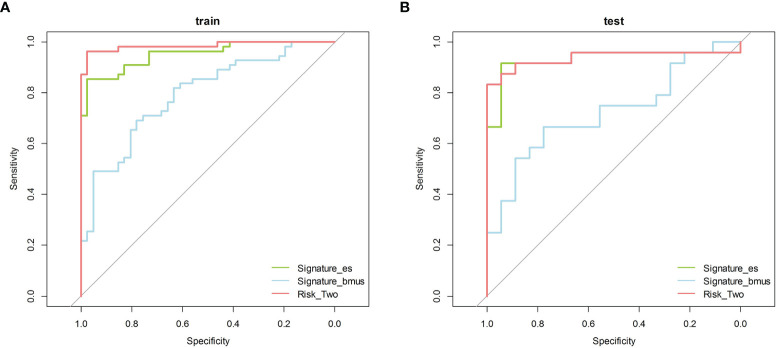
The ROC curves of the three models. ROC, receiver operating characteristic. **(A)** Training cohort **(B)** Test cohort.

**Figure 5:**
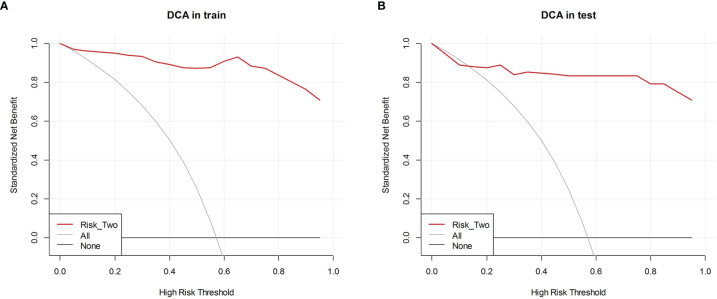
Decision curve analysis (DCA) of each model in predicting BRAF^V600E^ Mutations for papillary thyroid carcinoma (PTC). The vertical axis measures standardized net benefit. The horizontal axis shows the corresponding risk threshold. The DCA showed using the risk_two radiomics (red curve) derived in the present study to predict BRAF^V600E^ Mutations provided the greatest benefit. **(A)** DCA in training cohort **(B)** DCA in the test cohort.

According to the grayscale and elastic ultrasound images in the training dataset, logistic regression values were the best classifier. First, we evaluated the performances of the three models in the training dataset and then validated them in the test dataset. The AUCs of the grayscale, elasticity, grayscale, and elasticity combination prediction model in the training dataset was 0.792 (95% CI, 0.703−0.882), 0.952 (95% CI, 0.914−0.990), and 0.985 (95% CI, 0.965−1.000), respectively. In the test dataset, the AUCs were 0.725 (95% CI, 0.569−0.880), 0.931 (95% CI, 0.841−1.000), and 0.938 (95% CI, 0.851−1.000), respectively. The results showed that the elastic prediction model or the combination of the grayscale and elastic prediction model was better than the grayscale prediction model. The AUC, accuracy, sensitivity, and specificity of the three models are shown in **Table 3**. The ROC curves of the three models are shown in **Figure 4**. The decision curve analysis (DCA) was applied in determining the clinical usefulness of the radiomic by calculating the net benefits at different threshold values in the combined training and validation cohort. The DCA showed that radiometric provided a higher overall net benefit than the all or no treatment strategy (**Figure 5**).

**Table 3:** Performance of the sequences models.

Cohort	Model	AUC^a^	SEN^b^ (%)	SPE^c^ (%)	PPV^d^	NPV^e^	ACC^f^ (%)	Cutoff value
dataset	grayscale	0.792 (0.703–0.882)	69.1	78.0	80.9	65.3	72.9	0.372
	elasticity	0.952 (0.914–0.990)	85.5	97.6	97.9	83.3	90.6	0.469
	grayscale + elasticity	0.985 (0.965–1.000)	96.4	97.6	98.1	95.2	96.9	0.628
Test dataset	grayscale	0.725 (0.569–0.880)	54.2	83.3	81.2	57.7	66.7	0.372
	elasticity	0.931 (0.841–1.000)	83.3	94.4	95.2	81.0	88.1	0.469
	grayscale + elasticity	0.938 (0.851–1.000)	83.3	1.0	1.0	81.8	90.5	0.628

a AUC, area under the receiver operating characteristic curve.

b SEN, sensitivity.

c SPE, specificity.

d PPV, positive predictive value.

e NPV, negative predictive value.

f ACC, balanced accuracy.

## Discussion

At present, mutations in the BRAF^V600E^ gene positively correlate with high-risk clinicopathological factors of PTC such as extraglandular invasion, cervical lymph node metastasis, and tumor stage III-IV, suggesting that the BRAF^V600E^ mutation may reflect the degree of malignancy of PTC (17, 18). It plays a key role in the development and evolution of PTC and can be used as an important indicator to objectively judge the invasiveness of PTC. We thus believe that by detecting BRAF^V600E^ mutations and expanding the scope of surgical resection for cases with BRAF^V600E^ mutation, active treatment can be optimized thereby enabling a better prognosis. Because of its strong association with aggressive clinic pathological outcomes and serious molecular derangements in PTC, the BRAF mutation has emerged as a unique and valuable molecular marker in the management of PTC. The preoperative prediction of BRAF^V600E^ mutations in patients with PTC is therefore of great significance for guiding their clinical treatment.

Liu et al. predicted the BRAF^V600E^ mutation in patients with PTC using gray-scale ultrasound radiomics, demonstrating the feasibility of using the USR method for analysis (15). When compared with a single ultrasound imaging mode, USR can delve into details behind PTC ultrasound images to determine the temporal and spatial heterogeneity of PTC, indicating the relationship between image features and BRAF^V600E^ mutations. USR can therefore be used as a noninvasive imaging method for the preoperative evaluation of BRAF^V600E^ mutations in patients with PTC. In our study, we developed a preoperative, radiomic ultrasound model to improve the prediction of the BRAF^V600E^ mutation in patients with PTC. The AUC of gray-scale radiomic sequence model, elastic radiomic sequence model, and multi-sequence (grayscale combined with elastic ultrasound) radiomic model in the training dataset was 0.792, 0.952, 0.985 respectively, and in the test dataset were 0.725, 0.931, 0.938, respectively. Our study shows that both the elastic radiomic sequence model and the multi-sequence (grayscale combined with elastic ultrasound) sequence model can well predict the BRAF^V600E^ mutation in the training dataset and test dataset. Elasticity ultrasound can estimate the corresponding conditions inside PTC tissues *via* an ultrasonic imaging method combined with digital image processing or digital signal processing technology. Elastography reflects the relative hardness of the lesion and its surrounding tissues, while the hardness of PTC tissues is closely related to its internal pathological structure; therefore, real-time tissue elastography can evaluate the anatomical structure and biological characteristics of PTC.

Bojunga et al. showed that elasticity ultrasound can effectively distinguish between benign and malignant thyroid nodules (19), while Moon et al. found that PTC elastography findings with a degree of high hardness were associated with extrathyroidal invasion (20). These studies provide a new perspective for the ultrasound diagnosis of BRAF^V600E^ mutation-positive and negative PTC. Recent studies have shown that owing to the different arrangement and composition of cells, elasticity indices of parts of tissues are different (21, 22); the higher the malignancy of PTC, the greater the hardness on elasticity ultrasound imaging. There were significant differences in the elastic modulus or hardness between patients with and without BRAF^V600E^ mutations based on the ultrasound images. Elasticity could directly and quantitatively reflect the absolute hardness of BRAF^V600E^ mutation-positive and negative PTC and better reflect the pathological characteristics of tissues. Therefore, multimodal USR was used in this study to predict the BRAF^V600E^ mutation in patients with PTC. A significant association was revealed between ultrasound radiomic features and the BRAF^V600E^ mutation in PTC; thus, our analysis provides an alternative method that is noninvasive and convenient for the assessment of the BRAF^V600E^ mutation in patients. Using LASSO feature selection, 958 quantitative imaging features were reduced to 13 potential features (8 from grayscale and 5 from elasticity ultrasound) suitable for radiomic features analysis of the ultrasound datasets. Most of the selected imaging features were microscopic structural features of the tumor, including cellularity, and compression of the normal thyroid tissues by the tumor. We analyzed the relationship between radiomic features and the BRAF^V600E^ mutation and found 11 wavelet features, 1 first-order feature, and 1 texture feature that were significantly correlated with the BRAF^V600E^ mutation. According to the definitions of these texture features, these radiomic features can serve as a new tool for preoperative prediction of BRAF^V600E^ mutations in PTC patients.

There are some limitations to our study. First, this was a small sample retrospective study conducted at two institutions; thus, a selection bias may exist. In the future, we aim to conduct a multicenter study with a larger sample size. Second, only two ultrasound machines with similar parameters were used in this study to avoid the influence of equipment; however, parameter characteristics and patient-related factors may still affect the pixel intensity of the ultrasound images. Future studies should investigate several different types of ultrasonic instruments. Third, we focused on assessing the BRAF^V600E^ mutation in patients with PTC and did not study the gene in healthy individuals, which may have led to a selection bias. Finally, the lack of external validation data is a limitation of this study.

## Conclusions

Our preliminary study suggests that elasticity ultrasound, combined with gray-scale ultrasound imaging, has significant clinical value for predicting BRAF^V600E^ mutation in patients with PTC. This can provide clinicians with a more accurate and noninvasive diagnosis of the BRAF^V600E^ mutation before surgery thereby optimizing treatment outcomes.

## Data Availability Statement

The original contributions presented in the study are included in the article/supplementary material. Further inquiries can be directed to the corresponding authors.

## Ethics Statement

The studies involving human participants were reviewed and approved by Jiangsu University-affiliated peoples ethics committee. The patients/participants provided their written informed consent to participate in this study.

## Author Contributions

Conceptualization, Y-gW, F-jX, EA, XW, and X-qQ. Data curation, GZ, XB, W-zL, and SH. Formal analysis, F-jX, GZ, and W-zL. Funding acquisition, Y-gW, XW, S-dH, and X-qQ. Investigation, HX, Y-dW, and JZ. Methodology, EA, HS, and W-zL. Project administration, XW, S-dH, and X-qQ. Resources, Y-gW, F-jX, and SH. Software, HS, X-sB, and W-zL. Supervision, XW, SH, and X-qQ. Validation, Y-gW, F-jX, HX, Y-dW, and JZ. Visualization, Y-gW, F-jX, and X-sB. Writing – original draft, XW and X-qQ. Writing – review and editing, EA. All authors contributed to the article and approved the submitted version.

## Funding

The authors disclose the receipt of financial support for the research, authorship, and/or publication of this article from the Clinical Medicine Science and Technology Development Foundation of Jiangsu University (Project No. JLY2021001) and the Zhenjiang Commission of Science and Technology (Project No. SH2020046, Project No. SH2018050, and Project No. SH2018056). Guiding Project of Jiangsu Health Committee (Z2021071).

## Conflict of Interest

The authors declare that the research was conducted in the absence of any commercial or financial relationships that could be construed as a potential conflict of interest.

## Publisher’s Note

All claims expressed in this article are solely those of the authors and do not necessarily represent those of their affiliated organizations, or those of the publisher, the editors and the reviewers. Any product that may be evaluated in this article, or claim that may be made by its manufacturer, is not guaranteed or endorsed by the publisher.
